# Meta-Analysis of Randomized Controlled Trials of Atrial Fibrillation Ablation With Pulmonary Vein Isolation Versus Without

**DOI:** 10.1016/j.jacep.2019.05.012

**Published:** 2019-08

**Authors:** Arunashis Sau, James P. Howard, Sayed Al-Aidarous, João Ferreira-Martins, Becker Al-Khayatt, P. Boon Lim, Prapa Kanagaratnam, Zachary I. Whinnett, Nicholas S. Peters, Markus B. Sikkel, Darrel P. Francis, S.M. Afzal Sohaib

**Affiliations:** a National Heart and Lung Institute, Imperial College London, London, United Kingdom; b Department of Cardiology, Imperial College Healthcare NHS Trust, Hammersmith Hospital, London, United Kingdom; c Department of Cardiology, Royal Brompton and Harefield NHS Foundation Trust, London, United Kingdom; d Department of Cardiology, Royal Jubilee Hospital, Victoria, Canada; e Department of Cardiac Electrophysiology, Bart’s Heart Centre, St Bartholomew’s Hospital, London, United Kingdom; f Department of Cardiology, King George Hospital, Ilford, United Kingdom

**Keywords:** ablation, atrial fibrillation, pulmonary vein isolation, AF, atrial fibrillation, AT, atrial tachycardia, PVI, pulmonary vein isolation, CFAE, complex fractionated atrial electrograms, GP, ganglionated plexi, LVEF, left ventricular ejection fraction, PWI, posterior wall isolation, PAF, paroxysmal atrial fibrillation, PsAF, persistent atrial fibrillation, RCT, randomized controlled trial

## Abstract

**Objectives:**

This meta-analysis examined the ability of pulmonary vein isolation (PVI) to prevent atrial fibrillation in randomized controlled trials (RCTs) in which the patients not receiving PVI nevertheless underwent a procedure.

**Background:**

PVI is a commonly used procedure for the treatment of atrial fibrillation (AF), and its efficacy has usually been judged against therapy with anti-arrhythmic drugs in open-label trials. There have been several RCTs of AF ablation in which both arms received an ablation, but the difference between the treatment arms was inclusion or omission of PVI. These trials of an ablation strategy with PVI versus an ablation strategy without PVI may provide a more rigorous method for evaluating the efficacy of PVI.

**Methods:**

Medline and Cochrane databases were searched for RCTs comparing ablation including PVI with ablation excluding PVI. The primary efficacy endpoint was freedom from atrial fibrillation (AF) and atrial tachycardia at 12 months. A random-effects meta-analysis was performed using the restricted maximum likelihood estimator.

**Results:**

Overall, 6 studies (n = 610) met inclusion criteria. AF recurrence was significantly lower with an ablation including PVI than an ablation without PVI (RR: 0.54; 95% confidence interval [CI]: 0.33 to 0.89; p = 0.0147; I^2^ = 79.7%). Neither the type of AF (p = 0.48) nor the type of non-PVI ablation (p = 0.21) was a significant moderator of the effect size. In 3 trials the non-PVI ablation procedure was performed in both arms, whereas PVI was performed in only 1 arm. In these studies, AF recurrence was significantly lower when PVI was included (RR: 0.32; 95% CI: 0.14 to 0.73; p = 0.007, I^2^ 78%).

**Conclusions:**

In RCTs where both arms received an ablation, and therefore an expectation amongst patients and doctors of benefit, being randomized to PVI had a striking effect, reducing AF recurrence by a half.

In a randomized controlled trial (RCT), when a patient is randomized to an arm without a procedural intervention instead of an arm with a procedural intervention, both the patient and the patient’s medical care staff are inevitably aware that the patient has not had a procedure which other patients have had. This can easily lead to a lower threshold for reporting concerns and therefore undergoing follow-up tests. This in turn can lead to more adverse events becoming documented simply through the increased vigilance and opportunity for detection. This phenomenon has been termed “subtraction anxiety” (1). Blinding the patient to treatment allocation removes this phenomenon, but this can be challenging when invasive procedures are involved.

Pulmonary vein isolation (PVI) is the cornerstone of atrial fibrillation (AF) ablation procedures. However, its efficacy has been formally judged only compared to medical therapy in open-label trials (2). There is, however, a set of trials of AF ablation that allow us to establish the efficacy of pulmonary vein isolation relative to other methods of AF ablation. These trials have the additional advantage that, in the comparison arm where alternative ablation takes place, the patient has received a procedure and therefore the patients (and the doctors downstream) do not automatically feel subtraction anxiety. A particularly informative subset of these trials are those in which both arms received the same ablation procedure, except that one arm had PVI and the other did not. Those trials are informative of the incremental effect of PVI.

The present meta-analysis examined the ability of PVI to prevent AF in RCTs in which the patients who were not receiving PVI nevertheless underwent a procedure.

## Methods

This study carried out a meta-analysis of RCTs evaluating AF ablation, comparing a strategy involving PVI with a strategy not involving PVI.

### Search strategy

Four reviewers (A.S., S.A., J.F.M., and B.A.K.) searched the MEDLINE and Cochrane Central Register of Controlled Trials for trials of paroxysmal and persistent AF ablation. The searches were conducted on April 5, 2017. The search strategy details are shown in the Online Appendix. References of relevant studies were also searched manually. Abstracts and relevant full texts were independently screened by the reviewers. Disputes were resolved by consensus following discussion with another author (M.B.S.). The review protocol of this study was published in the PROSPERO (International Prospective Register of Systematic Reviews; CRD42018094577) database.

### Inclusion and exclusion criteria

RCTs of PVI versus non-PVI AF ablation were included. Studies were eligible if 1 arm included PVI while at least 1 other arm included left atrial ablation without PVI. Studies of partial PVI were not included.

### Endpoints

The primary endpoint was the proportion of patients who were free from AF and atrial tachycardia (AF/AT) after a single procedure. The event rate at 12 months was extracted. The 12-month time point was selected because this was the longest follow-up reported by most studies. Where not available, the nearest available time point was selected. The primary endpoint was electronically documented as an occurrence of AF or AT. Studies that reported only the rate of AF were included.

### Data extraction and analysis

Two authors (A.S. and J.H.) independently extracted the data. Analysis was based on the intention-to-treat outcomes from each study. The number of patients at risk, the total patients in the arm, and the number lost to follow-up were extracted from each arm. Data were also extracted regarding symptom scores where present. Where these data were not presented, they were calculated as described in the Online Appendix. A random-effects meta-analysis was performed using the restricted maximum likelihood estimator. Moderator variables were assessed using a mixed-effects meta-analytical model. The I^2^ statistic was used to assess heterogeneity. R software (3) with the Metafor feature (4) was used for all statistical analysis. The Cochrane risk-of-bias tool was used to assess included studies (5). Tests for publication bias were not performed because <10 trials were included for analysis (6). The Preferred Reporting Items for Systematic Reviews and Meta-Analyses guideline was used to report results (7). Values are mean ± SD, unless otherwise stated. Sensitivity analyses were performed, excluding each trial in turn.

## Results

MEDLINE and Cochrane database searches yielded a total of 2,544 studies (Online Figure 1). Seven studies met the inclusion criteria, 1 study was subsequently excluded. Therefore, 6 studies formed the final analysis and are detailed in Table 1 and Online Table 2.

**Table 1:** Study Characteristics

First Author (Ref. #)	Acronym	Year	n	Mean Age (yrs)	Sex (% Males)	Follow-Up (months)	PVI Arm Analyzed	Non-PVI Arm	Primary Endpoint	Endpoint Used for Analysis	%PsAF	Mean LA Diameter (mm)	Mean LVEF (%)	Hypertension (%)	Absence of AADs Mandated	Means of Detecting Recurrence
Chen et al. (11)	NA	2011	118	56.1	66.5	22.6 ± 6.4	PVI	CFAE	Freedom from AF/AT∗	Freedom from AF/AT	0	34.7	65.5	23	Yes	ECG and 24-h Holter 3 days and 1, 3, 6 and 12 months post-procedure. Additional testing if symptomatic
Di Biase et al. (23)	NA	2009	68	58.4	82.3	13.7 ± 2.2	PVI+CFAE	CFAE	Freedom from AF/AT	Freedom from AF/AT	0	46.2	55	35.7	No	Event recorder for 5 months with minimum 4 times per week recording. 48-h Holter at 3,6, 9, 12 and 15 months postprocedure
Katritsis et al. (24)	NA	2013	164	56	66	24	PVI+GP	GP	Freedom from AF/AT	Freedom from AF/AT	0	48.3	62.6	76.3	No	Monthly Holter for 2 yrs, transtelephonic transmission when symptomatic. 50% of patients also received an ILR.
Mamchur et al. (25)	NA	2014	79	56.6	60	16	PVI	GP	Freedom from AF	Freedom from AF∗	100	46.2	45.9	Not reported	No	24-h Holter, frequency not specified.
Atienza et al. (10)	RADAR-AF	2014	113	53.5	78.6	12	PVI	HFSA	Freedom from AF	Freedom from AF/AT	0	40	60	36.3	Yes	ECG and 48-h Holter at 3, 6, and 12 months post-procedure.
Verma et al. (26)	STAR AF	2010	68	57	74.3	12	PVI+CFAE	CFAE	Freedom from AF	Freedom from AF/AT/AFL	35.7	41.6	61.7	44.9	No	ECG and 48-h Holter at 3, 6, and 12 months post-procedure. External loop recorder and/or transtelephonic monitors used to confirm rhythm when symptomatic outside of follow-up visits.

AAD = antiarrhythmic drugs; AF = atrial fibrillation; AFL = atrial flutter; AT = atrial tachycardia; CFAE = complex fractionated atrial electrogram; ECG = electrocardiography; FIRM = focal impulse and rotor modulation; GP = ganglionated plexi; HFSA = high-frequency source ablation; ILR = implantable loop recorder; LA = left atrium; LVEF = left ventricular ejection fraction; NA = not applicable; PsAF = persistent AF; PVI = pulmonary vein isolation; RADAR-AF = Radiofrequency Ablation of Drivers of Atrial Fibrillation; STAR-AF = Substrate Versus Trigger Ablation for Reduction of Atrial Fibrillation Trial.

∗ Freedom from AF/AT was not reported.

The Cochrane risk-of-bias tool was used to assess trial quality (Online Table 1, Online Figure 2). The OASIS trial (8) was retracted and therefore not included in the main analysis (9).

A total of 796 patients were enrolled, of whom 496 were randomized to undergo PVI (with or without other ablation targets) and 300 to ablation not including PVI. Where possible, arms where the control therapy was included in the PVI arm were used in the analysis. A total of 310 patients undergoing PVI were therefore included in our main analysis. Across the 6 trials, mean follow-up was 18.5 months, with a total follow-up of 1225.5 patient years. Mean age was 56.4 years old, and 61.4% of participants were male. The RADAR-AF (Radiofrequency Ablation of Drivers of Atrial Fibrillation; NCT00674401) trial (10) enrolled patients with persistent AF (PsAF) and patients with paroxysmal AF (PAF). Only the PAF group was randomized to receive PVI versus no PVI, therefore, only this group was eligible for meta-analysis.

Chen et al. (11) randomized patients with PAF to receive PVI or complex fractionated atrial electrogram (CFAE) ablation. However, after randomization, if AF was inducible, patients received both PVI and CFAE ablation. A total of 24 of 60 in the PVI arm also had CFAE ablation, whereas 34 of 58 in the CFAE arm also had PVI. Therefore, this trial was analyzed as an intention-to-treat trial. Because more CFAE patients crossed over to PVI than PVI patients crossed over to CFAE, if any bias was introduced, it would be a tendency for PVI to appear artificially worse.

### Prevention of AF by PVI

Across all trials, the inclusion of PVI significantly reduced the occurrence of AF/AT (RR: 0.54; 95% confidence interval [CI]: 0.33 to 0.89; p = 0.0147) (Central Illustration). There was evidence of heterogeneity of this effect between trials (I^2^ = 79.7%). The total numbers of patients having occurrences of AF/AT after ablation were 90 of 306 (29.4%) with PVI and 147 of 284 (51.8%) without PVI.

**Central Illustration:**
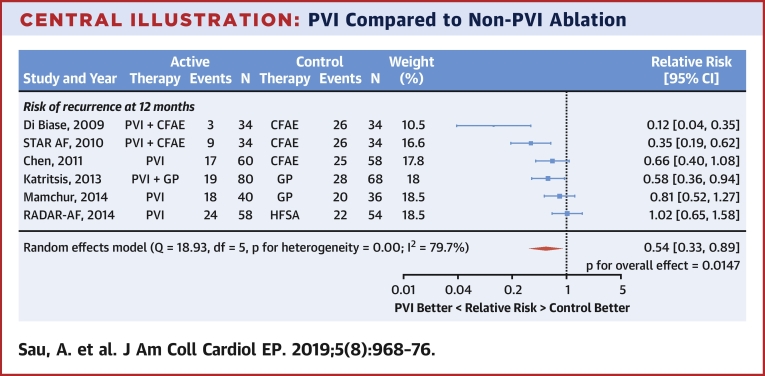
PVI Compared to Non-PVI Ablation PVI increases freedom from atrial arrhythmia by 46% compared to non-PVI ablation. CFAE = complex fractionated atrial electrogram; GP = ganglionated plexi; HFSA = high-frequency source ablation; PVI = pulmonary vein isolation; RADAR-AF = Radiofrequency Ablation of Drivers of Atrial Fibrillation; STAR-AF = Substrate Versus Trigger Ablation for Reduction of Atrial Fibrillation Trial.

**Figure 1:**
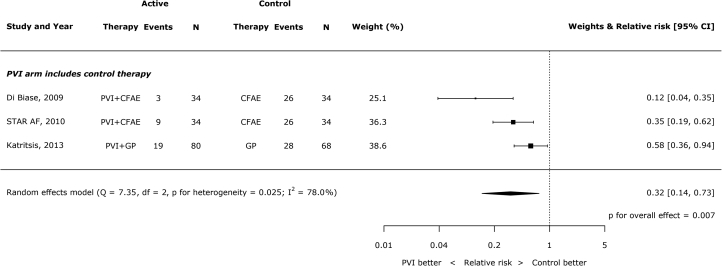
PVI Compared to Control Ablation Where the PVI Arm Also Included Control Therapy CFAE = complex fractionated atrial electrogram; GP = ganglionated plexi; PVI = pulmonary vein isolation; STAR-AF = Substrate Versus Trigger Ablation for Reduction of Atrial Fibrillation Trial.

There were 3 trials in which the only differences between arms were the presence of PVI. PVI significantly reduced the occurrences of AF/AT (RR: 0.32; 95% CI: 0.14 to 0.73; p = 0.007; I^2^ = 78%) (Figure 1). The total numbers of patients having recurrences of AF/AT after ablation were 31 of 148 (20.9%) with PVI and 80 of 136 (58.8%) without PVI.

### Type of AF and type of ablation

**Figure 2:**
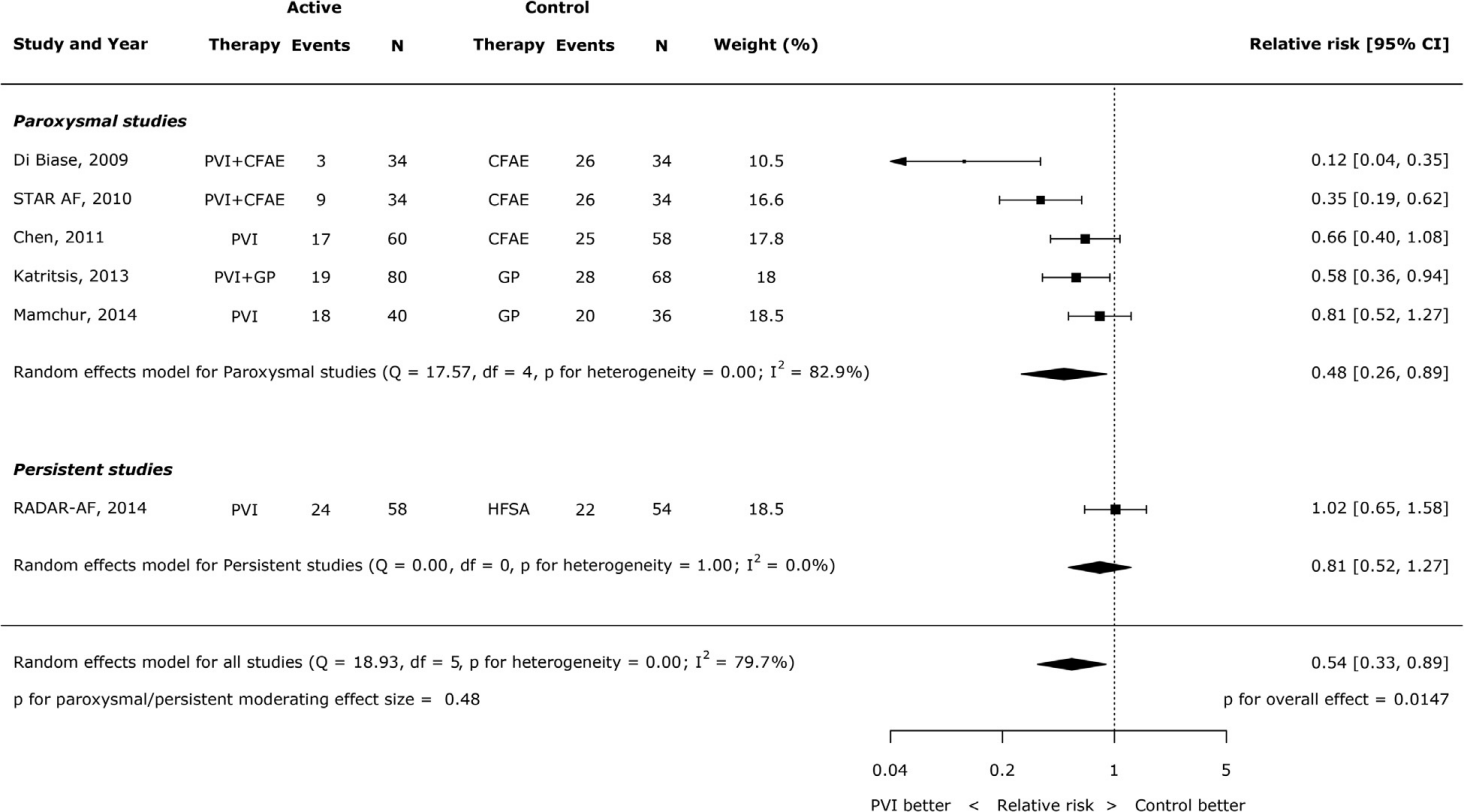
Impact of Type of AF (Persistent Versus Paroxysmal) on the Effect of PVI Versus Non-PVI Ablation on AF Recurrence AF = atrial fibrillation; other abbreviations as in Figure 1.

**Figure 3:**
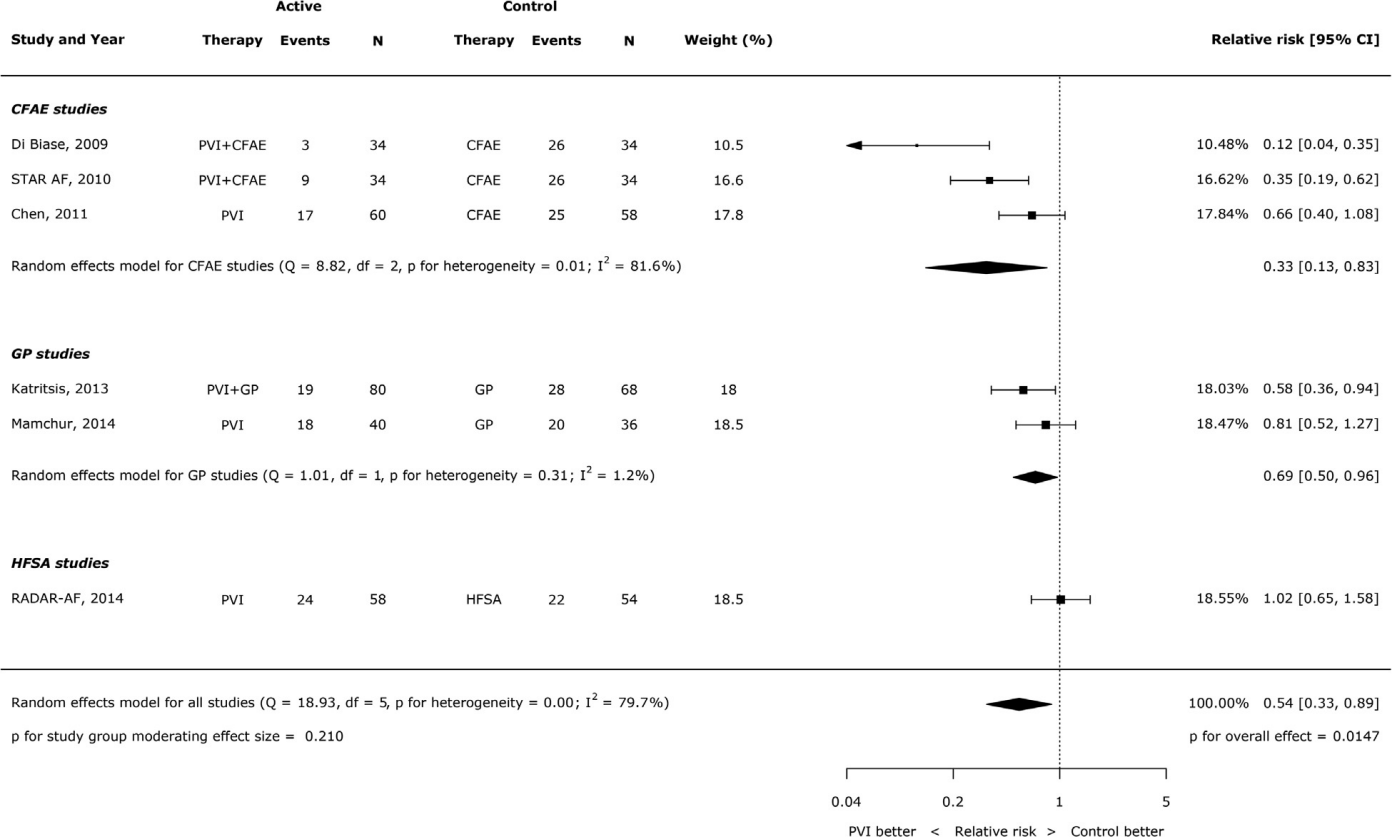
Effect of PVI Versus Non-PVI Ablation on AF Recurrence Stratified by the Control Arm Ablation Strategy RADAR-AF = Radiofrequency Ablation of Drivers of Atrial Fibrillation; other abbreviations as in Figure 1.

Type of AF (persistent versus paroxysmal) was not a significant moderator of effect size (p = 0.48) (Figure 2). For this analysis, patients in the STAR-AF (Substrate Versus Trigger Ablation for Reduction of Atrial Fibrillation trial; NCT00367757) trial were assigned to the paroxysmal group because only 35.7% of patients had persistent AF. The type of non-PVI ablation was also not a significant moderator of the effect size (p = 0.21). Of the control procedures used for comparison, there were only >200 patients suitable for analysis in the CFAE ablation trials. Among these patients, the relative risk of AF/AT with PVI versus without PVI was 0.33 (95% CI: 0.13 to 0.83) (Figure 3).

### Sensitivity analysis

A sensitivity analysis omitting each study in turn showed results similar to those in the primary analysis. In all cases there remained a reduction of AF/AT recurrences when PVI was included in the procedure. Five analyses had a significant reduction, with 1 showing only a trend to reduction (Online Figures 3 to 8, Online Table 3).

### Symptom scores

Only 2 trials used quality-of-life measurements before and after ablation. The STAR-AF trial reported using the Short-Form Health Survey (SF-36) (12). There was a significant improvement in mental component summary and physical component summary scores in both PVI ablation arms. Patients in the CFAE-only ablation arm had a significant improvement in physical component summary but not mental component summary scores. The RADAR-AF trial reported improvements in physical, mental, and sexual AF quality-of-life scores in both arms, but there were no significant differences between arms (7).

## Discussion

This analysis provides further evidence of efficacy of PVI in preventing AF/AT and, particularly, in comparison to alternative methods for ablating AF. The effect size is large when PVI is included as part of the ablation procedure, preventing between one-half and two-thirds of recurrences. This analysis offers an advantage over those which may compare PVI to medical therapy as patients in the control arm have all undergone an invasive ablation procedure.

Subtraction anxiety is a serious problem in unblinded trials of a procedure compared with medical therapy, where patients or doctors or both believe that a procedure is more efficacious than medical therapy. The use of a number of interventional procedures in cardiovascular medicine have been challenged following studies that have used a sham arm as the control. This procedure has included percutaneous coronary intervention for single-vessel coronary disease (13), renal denervation for hypertension (14), and patent foramen ovale (PFO) closure for migraine (15). In the unblinded FAME 2 (Fractional Flow Reserve versus Angiography for Multivessel Evaluation 2) (NCT01132495) trial (16), for instance, patients were randomized to not receive PCI despite the investigators having previously published their belief that the tests indicated that PCI was needed. Interestingly, shortly after discharge, there was an increase in the need for urgent revascularization because of symptoms in the absence of electrocardiography (ECG) or biomarker abnormalities. There was no accompanying early increase in myocardial infarctions or ECG abnormality events. This suggests that there may have been a bias (unconscious or otherwise) for the fact that a significant lesion that could easily have been treated was being left untreated. This could have encouraged patients to be more aware of symptoms and be more motivated to report them and, at the same time, could cause doctors to interpret reports with greater gravity. This would cause an artificial enhancement of not receiving the intervention or stent. Even in the case of a treatment with a well-documented prognostic benefit, biventricular pacing for heart failure, symptom improvement appeared greater in studies where patients with biventricular pacemakers were tested than in those receiving medical therapy alone versus studies where participants in the control arm were blinded and had a device but with no left ventricular lead pacing (17).

Some studies have questioned the success rates for PVI 18, 19. Part of the difficulty of measuring success comes from defining success (20). Varying intensities for monitoring documented AF and defining recurrence can attenuate what is defined as a successful procedure. Ultimately, the goal of AF ablation is reduction in symptom burden, and symptom perception is vulnerable to bias. Because the treatment is an invasive procedure, it too is susceptible to subtraction anxiety in trials. AF ablation has not undergone formal “sham”-controlled trials, and only a true placebo-controlled trial may give an accurate estimate of effect size. Although this analysis does not compare PVI to a true placebo arm, there are enough trials of suitable design here to assess the efficacy of the PVI component of AF ablation with a reduced vulnerability to inflation by subtraction anxiety. The merit of these 6 trials is that each of them had an invasive procedure in both arms. All patients therefore believed that they had undergone a substantial intervention, and all the doctors caring for them could see that the patients had received an ablation procedure.

Nevertheless, in some of these trials, the treatment arms differed in more than just the presence of PVI. For example, in 1 trial (11), the arm the present authors consider as the control arm had CFAE ablation (with no PVI), and the arm the present authors consider as the active arm had PVI (with no CFAE ablation). Such designs of trials are perfectly sensible for comparing 2 strategies but are imperfect for the specific purpose of obtaining an accurate estimate of the effect of PVI. This is because there is a possibility that the procedures in the non-PVI arm increase or decrease the recurrence of AF.

The closest we can get to a genuine control arm from these trials is the set of 3 in which the only difference between arms was the presence of PVI, but even this is not perfect (for the particular purpose of our analysis) because, if the background procedure present in both arms has an effect on AF, this will affect the scope for PVI to show its effect. For example, if the background procedure present in both arms is 100% effective in eliminating AF, there is no scope for PVI to be effective. At the other extreme, if the background procedure somehow damages the left atrium so that it becomes more able to sustain AF when exposed to a pulmonary vein trigger, then PVI may have an artificially enhanced efficacy in reducing AF as a between-arm comparison.

This subset of 3 trials show the relative risk for AF or AT of 0.32, which is, by any reckoning, an effective therapy. Even the full set of 6 trials shows a relative risk of 0.54, which is still effective. These data, as free of subtraction anxiety as is currently available, are supportive of PVI being the bedrock of any AF ablation procedure.

### Study limitations

This study addressed only AF/AT recurrences and not symptomatic relief. This is because only 2 of the trials reported symptom data and did so in different ways. The current study, therefore, was unable to relate freedom from arrhythmia to symptom regression. It is understandable that the trials focused on electrically documented events because the investigators would have been mindful of the vulnerability of purely symptom endpoints to unintended bias (subtraction anxiety). This is where a formal placebo-controlled trial of AF ablation would be most informative. Indeed, even the endpoint of ECG documented AF/AT has some vulnerability in the trials where patients reported symptoms and then had ECGs performed as a reaction to that. This is because patients or doctors in the arm with more subtraction anxiety would be more likely to be concerned about symptoms and therefore respond by performing an ECG or Holter monitoring and would therefore have more opportunity to pick up an episode of AF. A formal placebo-controlled trial would eliminate this problem.

The ablation procedures in the control arms may increase the risk of AT (21). This may artificially make PVI seem more effective, but this was one of the reasons why the present authors chose to analyze procedures where the alternative ablation was performed in both arms, so the associated risk of inducing AT was similar in both arms. Even in this circumstance, the efficacy of PVI was impressive. An understanding of AF has limitations, and we may not fully understand how the alternative ablation procedures may interact with PVI when it comes to modifying the cause of AF. This makes it difficult to conclusively say that what was measured was the pure effect of PVI.

There was a degree of heterogeneity between studies, and multiple factors likely contributed to this. These factors included the use of different control procedures, the presence or absence of the alternative ablation procedure in the PVI arm, the presence or absence of antiarrhythmic drugs, and the varying methods for detecting AF recurrence. Given that most of these factors were the same in each arm, these differences in methodology are unlikely to affect an estimate of treatment effect.

The existing trials of PVI compared to those of non-PVI ablation are small compared to some other trials in AF ablation and also have predominantly male participants; however, there is currently no better evidence to use in this respect. The larger trials in this analysis show smaller treatment effects than the smaller trials. Larger effect sizes accumulated from the smaller studies may potentially lead to an overestimation of the efficacy of PVI in this analysis.

The efficacy of complete versus selective PVI has been studied (22). That analysis suggested that isolation of arrhythmogenic PVs alone is comparable to empirical isolation of all PVs. As the aim of both arms was to isolate pulmonary vein triggers for AF, it was not included in this analysis.

## Conclusions

Analysis of the present study provides an estimate of the efficacy of PVI in preventing AF/AT. This is a reduction of one-half and perhaps even two-thirds if only the purest trials are considered. A true placebo-controlled trial of PVI versus placebo PVI (and no other procedure) may show an even larger efficacy because there would be no background efficacy in the control arm. It remains unknown how these convincing reductions in electrically documented AF would relate to symptom regression, as the correspondence between arrhythmia and symptoms is imperfect. A placebo-controlled RCT, as is routine for pharmacotherapy, would be the ideal method of testing this.

Perspectives **COMPETENCY IN MEDICAL KNOWLEDGE:** This meta-analysis shows that PVI reduces AF recurrence by at least one-half compared to alternative ablation targets. Although alternative methods of AF ablation have been proposed, the present study demonstrated that, for now, PVI remains a cornerstone of AF ablation.

**TRANSLATIONAL OUTLOOK:** AF ablation has not undergone a comparison trial relative to an invasive placebo control. The correspondence between arrhythmia and symptoms is imperfect; therefore, it is unclear how reductions in AF burden, which PVI appears to do effectively, relates to symptom regression. A placebo (sham)-controlled RCT would be the ideal method for testing this.
