# Supplementary material for: Meta-Analysis of Randomized Controlled Trials of Atrial Fibrillation Ablation With Pulmonary Vein Isolation Versus Without
Source: JACC Clin Electrophysiol. 2019 Aug;5(8):968–76. doi: 10.1016/j.jacep.2019.05.012 (PMC6709782; doi:10.1016/j.jacep.2019.05.012)

Meta-analysis of randomized controlled trials of atrial fibrillation ablation with pulmonary vein isolation versus without

Online Appendix

Search strategy

Searches run between 5th and 19th April 2017
Searched from database inception

PubMed (Paroxysmal AF)

1. paroxysmal atrial fibrillation ablation

PubMed (Persistent AF)

1. (persistent or non-paroxysmal or non paroxysmal)
2. atrial fibrillation
3. ablation
4. Cochrane highly Sensitive Search Strategy for identifying randomized trials (http://handbook-5-1.cochrane.org)

Cochrane CENTRAL (Paroxysmal AF)

1. paroxysmal atrial fibrillation ablation

Cochrane CENTRAL (Persistent AF)

1. (persistent or non-paroxysmal or non paroxysmal)
2. atrial fibrillation
3. ablation

Data extraction

Chen et al

Patients randomized to PVI or CFAE, if AF still inducible after initial therapy patients cross-over to have both PVI and CFAE ablation. For our analysis, we analysed on an intention-to-treat basis. More patients in the CFAE arm crossed over than in the PVI arm.

Outcomes at 22.6 ± 6.4 months given in text.
PVI, n = 60, events = 17

CFAE, n = 58, events = 25

Katritsis et al

KM curve used to calculate % recurrences at 12 months. Numbers at risk at 15 months of follow up used as a conservative estimate to determine the approximate number of patients that would have still been in the study at 12 months.
Number at risk taken from figure 4 of the manuscript, events calculated using KM curve (using % recurrences at 12 month time point (figure 4 of manuscript).
PVI + GP, number at risk = 61. Freedom from atrial arrhythmia from KM curve = 76% (at 12 months), therefore n = 61÷ 0.76 = 80, events therefore = 80 – 61 = 19

GP, number at risk = 40. Freedom from atrial arrhythmia from KM curve = 58.7% (at 12 months), therefore n = 40 ÷ 0.587 = 68, events therefore = 68 – 40 = 28

Mamchur et al

Outcomes at 12 months used. % freedom from AF taken from KM curve, figure 6 of the manuscript.
PVI, n = 42. 2 patients censored, therefore n at 12 months = 40.
Freedom from atrial arrhythmia from KM curve = 55.1%.
40 x 55.1% = 22
Therefore events = 40-22 = 18

GP, n = 37. 1 patient censored, therefore n at 12 months = 36
Freedom from atrial arrhythmia from KM curve = 45.5%.

36 x 45.5% = 16
Therefore events = 36-16 = 20

STAR AF
Outcomes at 12 months used, data taken from Figure 3B
PVI + CFAE, number at risk = 25. n = 34. Therefore events = 34-25 = 9

CFAE, number at risk = 8. n = 34. Therefore events = 34-8 = 26

RADAR-AF
Number at risk taken from figure 5
PVI
1 patient lost to follow up, therefore n = 58
Number at risk at 12 months = 34
Events therefore = 58-34 = 24

HFSA
2 patient lost to follow up, therefore n = 54
Number at risk at 12 months = 32
Events therefore = 54-32 = 22

Di Biase et al
Outcomes at 12 months reported in table 4 of manuscript

PVI+CFAE, n = 34, events = 3
CFAE, n = 34, events = 26

Table 1

Trial	Random sequence generation	Allocation concealment	Blinding of participants and personnel.	Blinding of outcome assessment.	Incomplete outcome data.	Selective reporting	Other bias
**Chen et al**	Low risk – sealed envelopes	Unclear<br>Not specified	Low risk<br>Participants blinded, but personnel not blinded	Unclear<br>Not specified	Low risk<br>PVI: 1 excluded in due to acute procedural failure	Low risk<br>All endpoints on CT.gov reported	Large crossover to both PVI and CFAE ablation, with small numbers remaining in PVI or CFAE only groups.
**Di Biase et al**	Low risk – 'web-based centralized control program' permuted block strategy	Unclear – 'Web-based'	High risk - unblinded	Unclear<br>Not specified	Low risk – no loss to follow up	High risk<br>Not registered on CT.gov	
**Katritsis et al**	Low risk – computer-generated list of random numbers	Low risk<br>'Allocation concealment was safeguarded by ensuring that allocation was obtained by computer output	High risk - unblinded	Low risk – outcome assessors blinded to treatment arm	Low risk – no loss to follow up	High risk<br>Endpoint of quality of life not reported	

		after the patients had consented.’					
**Mamchur et al**	Unclear – not specified	Unclear – not specified	High risk - unblinded	Unclear<br>Not specified	Low risk<br>Censored outcomes:<br>ePVI: 7 (17%)<br>PVI: 6 (14%)<br>GP: 3 (8%)	High risk<br>Not registered on CT.gov	
**RADAR-AF**	Low risk – “web-based system and was balanced at each site”	Unclear – not specified	Low risk<br>Participants blinded, but personnel not blinded	Low risk – outcome assessors blinded to treatment arm	Low risk –<br>PVI – 1 lost to follow-up<br>HFSA – 1 excluded due to protocol violation, 1 withdrawn consent	Low risk<br>All endpoints on CT.gov reported however quality of life reported by AF-QOL questionnaire, while SF-36 pre-specified on CT.gov	
**STAR AF**	Low risk – “sequentially numbered, opaque, sealed envelopes”	Low risk – “sequentially numbered, opaque, sealed envelopes”	Low risk<br>Participants blinded, but personnel not blinded	Unclear<br>Not specified	Low risk – no loss to follow up	Low risk<br>All endpoints on CT.gov reported (quality of life reported in separate manuscript[12])	Sponsored by St Jude Medical
**Overall**	Low risk – most trials	Unclear risk, as most studies did not specify	High risk, more than half of the studies were	Unclear risk, not specified in most studies	Low risk – all trials judged to be low risk	Low risk – selective reporting of	

	judged to be low risk	means of safeguarding allocation concealment	unblinded for participants and personnel			endpoints may have significant impact. However the outcome of interest (freedom from AF/AT) was reported by 6 of the 7 studies.	

Risk of bias
PVI, pulmonary vein isolation; CFAE, complex fractionated atrial electrogram; CT.gov, www.clinicaltrials.gov

Table 2

Author	AAD use	Non PVI ablation technique	Thromboembolic risk	Duration of AF/AT required to define as recurrence	Method for ablation of ATs arising during the index procedure	Ablation endpoint for PVI	Ablation endpoint for control arm	complications
Chen [10]	Amiodarone stopped for 2 months pre ablation, other AADs stopped for 5 half lives. AADs reinstated at 3 months and continued for 3 months post ablation.	CFAE ablation, automated detection and ablation at relevant sites	Mean CHADS-2 score<br><br>PVI 0.49 ± 0.56<br>CFAE 0.47 ± 0.62	>30 secs	AT mapped and ablated accordingly, details not specified.	Complete disappearance of PV potentials or dissociated PV potentials with LA electrograms.	Elimination of the areas with CFAE as defined by the electroanatomical mapping	Not separated by ablation group.<br>4 major events, 1 perforation and subsequent tamponade. 1 massive pericardial effusion. 2 haemothoraces.
Di Biase [14]	Amiodarone stopped for 6 months pre ablation, other AADs stopped for 5 half lives.	CFAE areas ablated until CFAEs completely eliminated	Not described	>60 secs	AT mapped and ablated accordingly, details not specified.	Local elimination of all the pulmonary vein potentials along the antra or inside the veins (entry and exit block). Achieved in all patients.	Complete elimination of the CFAEs potentials. Achieved in all patients	No major complications

	Patients discharged on previous AADs except amiodarone. All AADs discontinued 2 months after procedure if no recurrences present							
Katritsis [15]	All patients received beta blockers (unless contraindicated. Strategy with other AADs not described	PVI + GP group underwent PVI by anatomic ablation of the 4 major left atrial GP ensuring PVI line was in continuation with GP ablation sites.	Not described	>30 secs	Not described	Entrance and exit block as determined with the use of a circular mapping catheter.	Not described	1 cardiac tamponade in the PVI group

Mamchur [16]	All patients received amiodarone pre-procedure (duration not specified)	Not described	Not described	Not specified	Not described	Entrance and exit block confirmed electrophysiologyically without the use of adenosine	Not described	Not described

Atienza[7]	AADs stopped for 5 half lives pre-procedure except amiodarone. AADs continued for 2 months post procedure.	HFSA performed by dominant frequency (DF) mapping (by use of automated algorithm). HFS ablated. If AF not terminated after LA HFSA DF mapped from RA and CS, maximum of 3-4 HFS per chamber were ablated (4 in LA, 3 in RA and CS).	PVI arm: CHADS 2 = 0 in 62%, 1 in 33% and ≥2 in 5%<br><br>HFSA arm: CHADS 2 = 0 in 53%, 1 in 40% and ≥2 in 7%	>30 secs	AT or AFL could be mapped and ablated at the discretion of the operator	Confirmation of entrance block	1) elimination of all HFS or conversion to SR; and 2) noninducibility of AF post-ablation	Major complications: 2 tamponade in PVI arm, 1 in HFSA arm.

Verma[17]	Amiodarone stopped for 8 weeks pre ablation, other AADs stopped for 5 half lives. AADs discontinued 2 months post procedure	PVI performed first, then CFAE mapping using automated software. CFAE sites defined as CL <120ms targeted for ablation.	Not described	>30 secs	AT or AFL could be mapped and ablated or cardioverted at the discretion of the operator	Electrical isolation confirmed with circular catheter. 30 min wait time to confirm isolation. Achieved in 94% of patients (in both PVI and PVI+CFE arm)	All CFAE sites ablated or AF non-inducible. AF non inducible in 68%.	Major complications: Tamponade, 1 in CFAE arm, 1 in PVI arm.

Further study specific data

Participant bleeding risk and risk factor management not described in any of the 7 studies.

AAD; ant-arrhythmic drug, CL; cycle length, CFAE; complex fractionated atrial electrogram, HFSA; high frequency source ablation.

Table 3
Summary table of sensitivity analyses

Trial excluded	Relative risk	P for overall effect
STAR AF	0.59 (0.34-1.03)	0.062
RADAR AF	0.47 (0.28-0.80)	0.006
Mamchur et al	0.48 (0.26-0.89)	0.02
Katritsis et al	0.52 (0.27-0.99)	0.047
Di Biase et al	0.66 (0.47-0.92)	0.015
Chen et al	0.50 (0.26-0.96)	0.037

Figure 1

PRISMA Flow chart

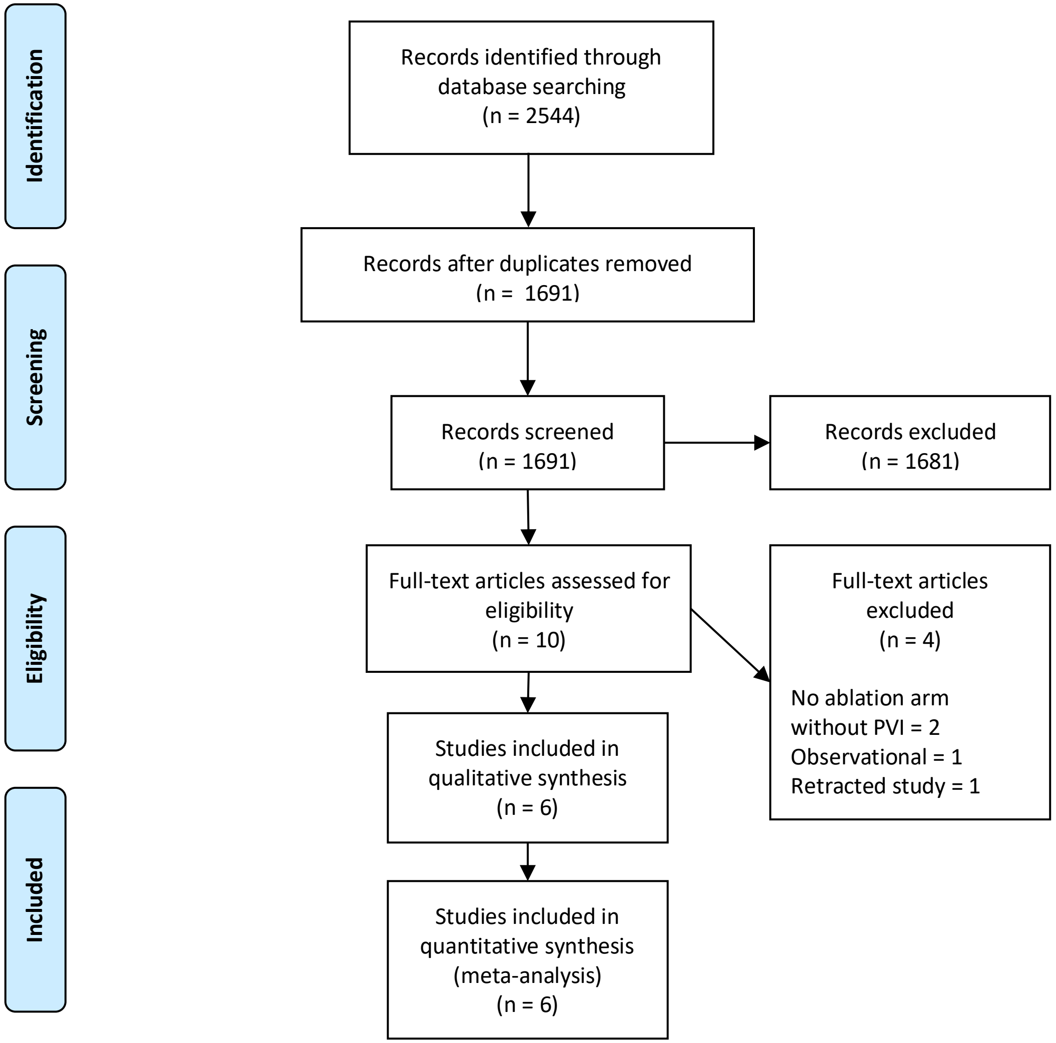

Figure 2

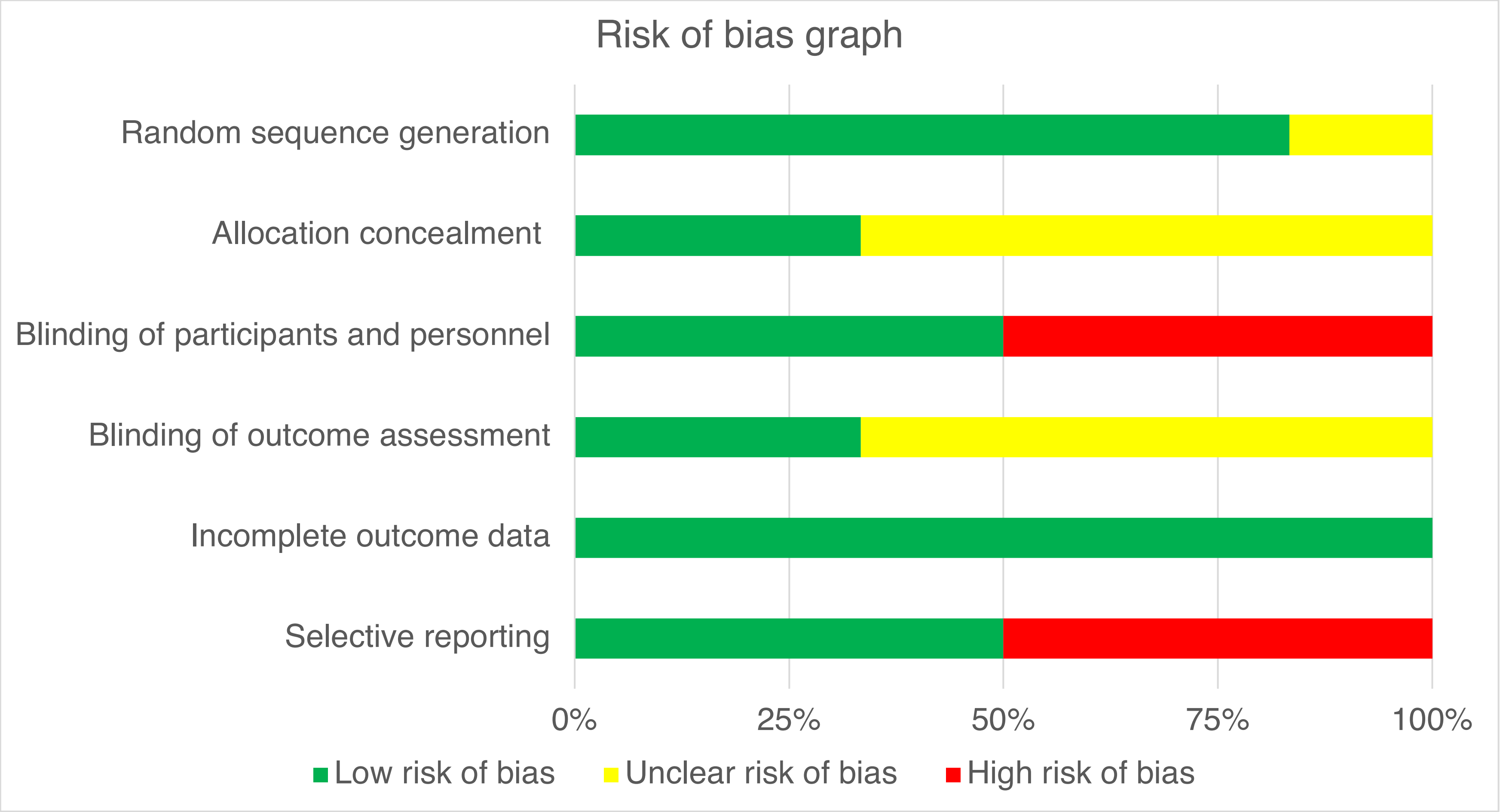
Risk of bias graph
Random sequence generation
Allocation concealment
Blinding of participants and personnel
Blinding of outcome assessment
Incomplete outcome data
Selective reporting
0%
25%
50%
75%
100%
Low risk of bias
Unclear risk of bias
High risk of bias

Sensitivity analyses

Figure 3

Effect of PVI vs. Non-PVI ablation on AF recurrence, excluding STAR AF

Study and Year	Active Therapy	Active Events	Active N	Control Therapy	Control Events	Control N	Weight (%)	Relative risk [95% CI]
Risk of AF recurrence at 12 months								
Di Biase, 2009	PVI+CFAE	3	34	CFAE	26	34	12.9	0.12 [0.04, 0.35]
Chen, 2011	PVI	17	60	CFAE	25	58	21.3	0.66 [0.40, 1.08]
Katritsis, 2013	PVI+GP	19	80	GP	28	68	21.6	0.58 [0.36, 0.94]
Mamchur, 2014	PVI	18	40	GP	20	36	22	0.81 [0.52, 1.27]
RADAR-AF, 2014	PVI	24	58	HFSA	22	54	22.1	1.02 [0.65, 1.58]
Random effects model (Q = 14.16, df = 4, p for heterogeneity = 0.01; I^2 = 81.6%)								0.59 [0.34, 1.03]

p for overall effect = 0.0628

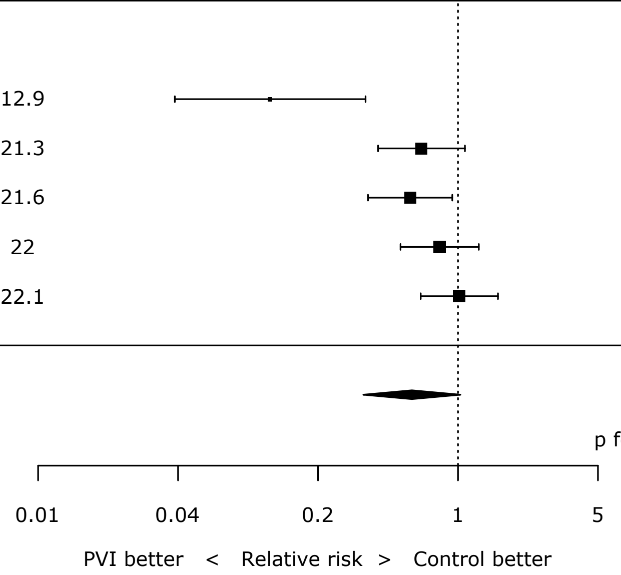

Figure 4

Effect of PVI vs. Non-PVI ablation on AF recurrence, excluding RADAR-AF

Study and Year	Active Therapy	Active Events	Active N	Control Therapy	Control Events	Control N	Weight (%)	Relative risk [95% CI]
Risk of AF recurrence at 12 months								
Di Biase, 2009	PVI+CFAE	3	34	CFAE	26	34	12.6	0.12 [0.04, 0.35]
STAR AF, 2010	PVI+CFAE	9	34	CFAE	26	34	20.4	0.35 [0.19, 0.62]
Chen, 2011	PVI	17	60	CFAE	25	58	22	0.66 [0.40, 1.08]
Katritsis, 2013	PVI+GP	19	80	GP	28	68	22.2	0.58 [0.36, 0.94]
Mamchur, 2014	PVI	18	40	GP	20	36	22.8	0.81 [0.52, 1.27]
Random effects model (Q = 13.51, df = 4, p for heterogeneity = 0.01; I^2 = 76.8%)								0.47 [0.28, 0.80]

p for overall effect = 0.0055

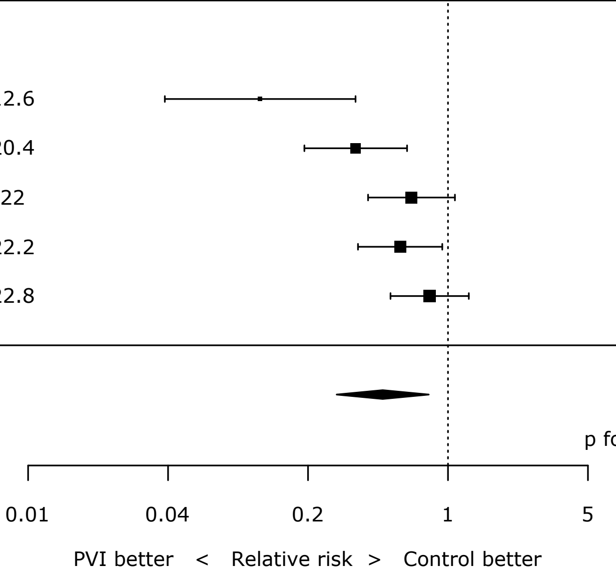

Figure 5

Effect of PVI vs. Non-PVI ablation on AF recurrence, excluding Mamchur et al

Study and Year	Active Therapy	Active Events	Active N	Control Therapy	Control Events	Control N	Weight (%)	Relative risk [95% CI]
Risk of AF recurrence at 12 months								
Di Biase, 2009	PVI+CFAE	3	34	CFAE	26	34	13.9	0.12 [0.04, 0.35]
STAR AF, 2010	PVI+CFAE	9	34	CFAE	26	34	20.4	0.35 [0.19, 0.62]
Chen, 2011	PVI	17	60	CFAE	25	58	21.6	0.66 [0.40, 1.08]
Katritsis, 2013	PVI+GP	19	80	GP	28	68	21.8	0.58 [0.36, 0.94]
RADAR-AF, 2014	PVI	24	58	HFSA	22	54	22.3	1.02 [0.65, 1.58]
Random effects model (Q = 17.57, df = 4, p for heterogeneity = 0.00; I^2 = 82.9%)								0.48 [0.26, 0.89]

p for overall effect = 0.0204

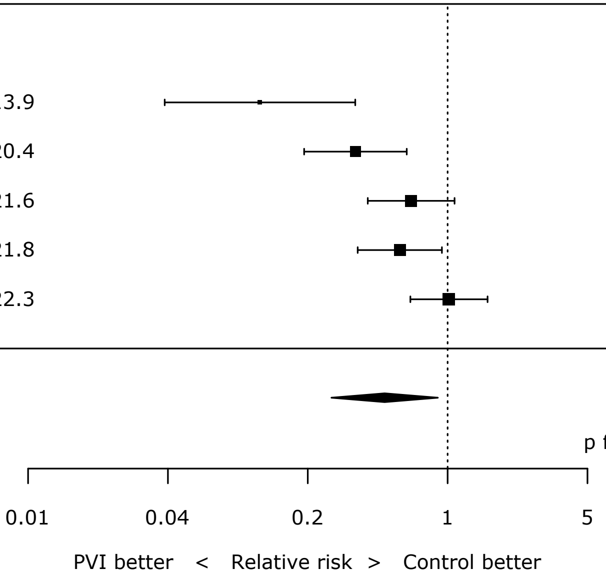

Figure 6

Effect of PVI vs. Non-PVI ablation on AF recurrence, excluding Katritsis et al

Study and Year	Active Therapy	Active Events	Active N	Control Therapy	Control Events	Control N	Weight (%)	Relative risk [95% CI]
Risk of AF recurrence at 12 months								
Di Biase, 2009	PVI+CFAE	3	34	CFAE	26	34	14.4	0.12 [0.04, 0.35]
STAR AF, 2010	PVI+CFAE	9	34	CFAE	26	34	20.4	0.35 [0.19, 0.62]
Chen, 2011	PVI	17	60	CFAE	25	58	21.4	0.66 [0.40, 1.08]
Mamchur, 2014	PVI	18	40	GP	20	36	21.9	0.81 [0.52, 1.27]
RADAR-AF, 2014	PVI	24	58	HFSA	22	54	21.9	1.02 [0.65, 1.58]
Random effects model (Q = 18.72, df = 4, p for heterogeneity = 0.00; I^2 = 85.4%)								0.52 [0.27, 0.99]

p for overall effect = 0.0469

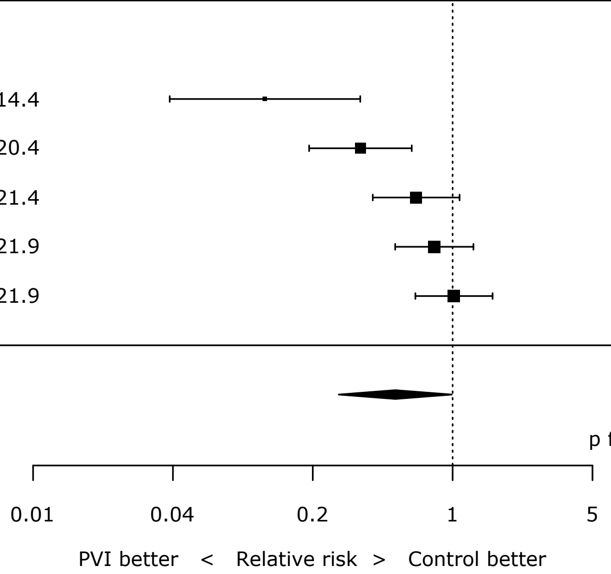

Figure 7
Effect of PVI vs. Non-PVI ablation on AF recurrence, excluding Di Biase et al

	Active			Control				
**Study and Year**	**Therapy**	**Events**	**N**	**Therapy**	**Events**	**N**	**Weight (%)**	**Relative risk [95% CI]**
Risk of AF recurrence at 12 months								
STAR AF, 2010	PVI+CFAE	9	34	CFAE	26	34	16.8	0.35 [0.19, 0.62]
Chen, 2011	PVI	17	60	CFAE	25	58	19.7	0.66 [0.40, 1.08]
Katritsis, 2013	PVI+GP	19	80	GP	28	68	20.2	0.58 [0.36, 0.94]
Mamchur, 2014	PVI	18	40	GP	20	36	21.5	0.81 [0.52, 1.27]
RADAR-AF, 2014	PVI	24	58	HFSA	22	54	21.7	1.02 [0.65, 1.58]
Random effects model ($Q = 9.20$, df = 4, p for heterogeneity = 0.06; $I^2 = 57.0\%$)								0.66 [0.47, 0.92]
								p for overall effect = 0.0151

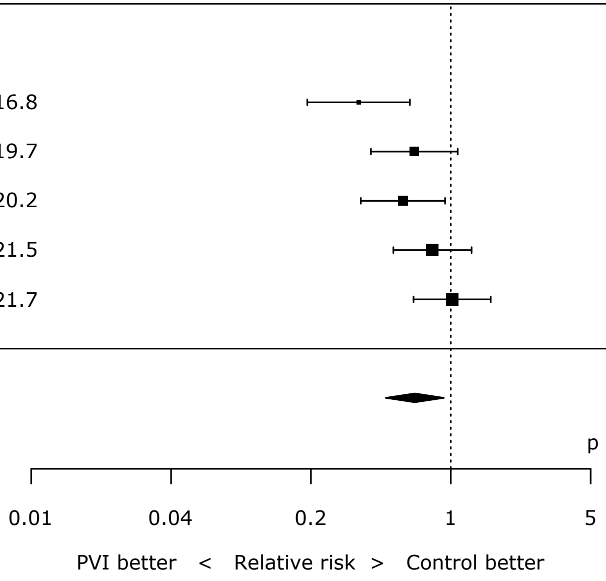

Figure 8

Effect of PVI vs. Non-PVI ablation on AF recurrence, excluding Chen et al

Study and Year	Active Therapy	Active Events	Active N	Control Therapy	Control Events	Control N	Weight (%)	Relative risk [95% CI]
Risk of AF recurrence at 12 months								
Di Biase, 2009	PVI+CFAE	3	34	CFAE	26	34	14.3	0.12 [0.04, 0.35]
STAR AF, 2010	PVI+CFAE	9	34	CFAE	26	34	20.3	0.35 [0.19, 0.62]
Katritsis, 2013	PVI+GP	19	80	GP	28	68	21.5	0.58 [0.36, 0.94]
Mamchur, 2014	PVI	18	40	GP	20	36	21.9	0.81 [0.52, 1.27]
RADAR-AF, 2014	PVI	24	58	HFSA	22	54	22	1.02 [0.65, 1.58]
Random effects model (Q = 18.92, df = 4, p for heterogeneity = 0.00; I^2 = 85.2%)								0.50 [0.26, 0.96]

p for overall effect = 0.0366

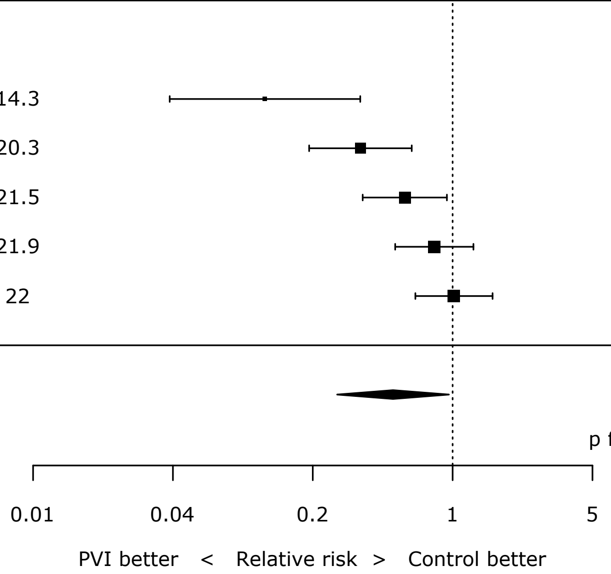

Sensitivity analysis including OASIS

A sensitivity analysis was performed which includes the retracted OASIS trial. Follow up duration in OASIS was 12 ± 7 months. We modelled this as a normal distribution and found 80.4% of patients passed 6 months follow up, the following analyses therefore use the event rate at 6 months.

Figure 9

Effect of PVI vs. Non-PVI ablation on AF recurrence at 6 months including the retracted trial OASIS

Study and Year	Active Therapy	Active Events	Active N	Control Therapy	Control Events	Control N	Weight (%)		Relative risk [95% CI]
CFAE studies									
Di Biase, 2009	PVI+CFAE	2	34	CFAE	14	34	2.9	2.86%	0.14 [0.04, 0.58]
STAR AF, 2010	PVI+CFAE	6	34	CFAE	19	34	9.1	9.14%	0.32 [0.14, 0.69]
Chen, 2011	PVI	17	60	CFAE	25	58	22.6	22.60%	0.66 [0.40, 1.08]
Random effects model for CFAE studies (Q = 5.48, df = 2, p for heterogeneity = 0.06; I^2 = 64.0%)									0.37 [0.17, 0.83]
GP studies									
Katritsis, 2013	PVI+GP	18	81	GP	23	78	19.9	19.86%	0.75 [0.44, 1.28]
Mamchur, 2014	PVI	9	41	GP	9	37	8.6	8.59%	0.90 [0.40, 2.03]
Random effects model for GP studies (Q = 0.13, df = 1, p for heterogeneity = 0.72; I^2 = 0.0%)									0.80 [0.51, 1.24]
FIRM studies									
OASIS, 2016	PVI+FIRM	13	34	FIRM	12	23	16.8	16.79%	0.73 [0.41, 1.31]
HFSA studies									
RADAR-AF, 2014	PVI	18	58	HFSA	19	55	20.2	20.16%	0.90 [0.53, 1.52]
Random effects model for all studies (Q = 10.17, df = 6, p for heterogeneity = 0.12; I^2 = 0.0%)								100.00%	0.67 [0.53, 0.85]
p for study group moderating effect size = 0.350									p for overall effect = 0.0011

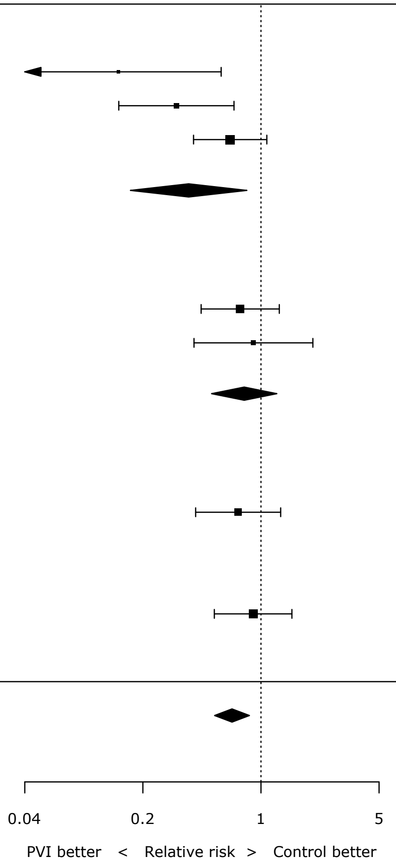

Figure 10
Effect of PVI vs. Non-PVI ablation on AF recurrence at 6 months excluding the retracted trial OASIS

Study and Year	Active Therapy	Active Events	Active N	Control Therapy	Control Events	Control N	Relative risk [95% CI]
CFAE studies							
Chen, 2011	PVI	17	60	CFAE	25	58	0.66 [0.40, 1.08]
Di Biase, 2009	PVI+CFAE	2	34	CFAE	14	34	0.14 [0.04, 0.58]
STAR AF, 2010	PVI+CFAE	6	34	CFAE	19	34	0.32 [0.14, 0.69]
Random effects model for CFAE studies (Q = 5.48, df = 2, p for heterogeneity = 0.06; I^2 = 64.0%)							0.37 [0.17, 0.83]
GP studies							
Katritsis, 2013	PVI+GP	18	81	GP	23	78	0.75 [0.44, 1.28]
Mamchur, 2014	PVI	9	41	GP	9	37	0.90 [0.40, 2.03]
Random effects model for GP studies (Q = 0.13, df = 1, p for heterogeneity = 0.72; I^2 = 0.0%)							0.80 [0.51, 1.24]
HFSA studies							
RADAR-AF, 2014	PVI	18	58	HFSA	19	55	0.90 [0.53, 1.52]
Random effects model for all studies (Q = 10.07, df = 5, p for heterogeneity = 0.07; I^2 = 38.5%)							0.63 [0.44, 0.89]
p for interaction between study group and effect size = 0.206							p for overall effect = 0.009

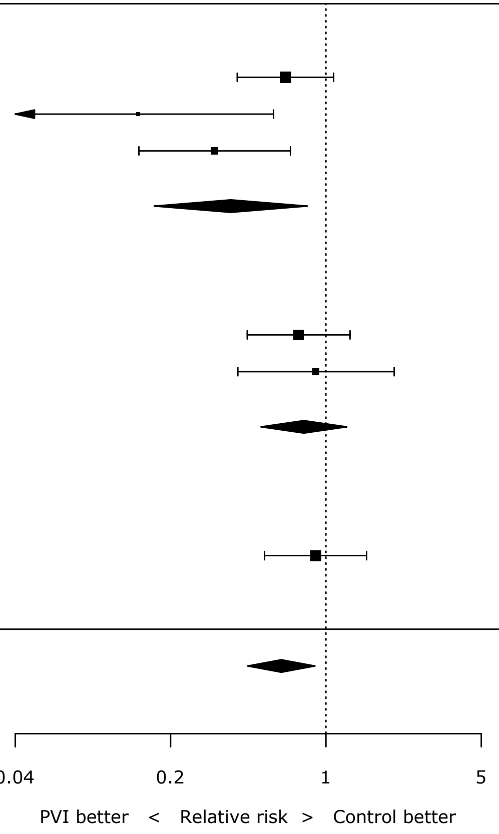

Figure 11

Effect of PVI vs. Non-PVI ablation on AF recurrence at 6 months including the retracted trial OASIS, excluding Chen et al

Study and Year	Active Therapy	Active Events	Active N	Control Therapy	Control Events	Control N	Relative risk [95% CI]
CFAE studies							
Di Biase, 2009	PVI+CFAE	2	34	CFAE	14	34	0.14 [0.04, 0.58]
STAR AF, 2010	PVI+CFAE	6	34	CFAE	19	34	0.32 [0.14, 0.69]
Random effects model for CFAE studies (Q = 0.93, df = 1, p for heterogeneity = 0.33; I^2 = 0.0%)							0.26 [0.13, 0.52]
GP studies							
Katritsis, 2013	PVI+GP	18	81	GP	23	78	0.75 [0.44, 1.28]
Mamchur, 2014	PVI	9	41	GP	9	37	0.90 [0.40, 2.03]
Random effects model for GP studies (Q = 0.13, df = 1, p for heterogeneity = 0.72; I^2 = 0.0%)							0.80 [0.51, 1.24]
FIRM studies							
OASIS, 2016	PVI+FIRM	13	34	FIRM	12	23	0.73 [0.41, 1.31]
HFSA studies							
RADAR-AF, 2014	PVI	18	58	HFSA	19	55	0.90 [0.53, 1.52]
Random effects model for all studies (Q = 10.16, df = 5, p for heterogeneity = 0.07; I^2 = 41.2%)							0.64 [0.44, 0.92]
p for interaction between study group and effect size = 0.028							p for overall effect = 0.017

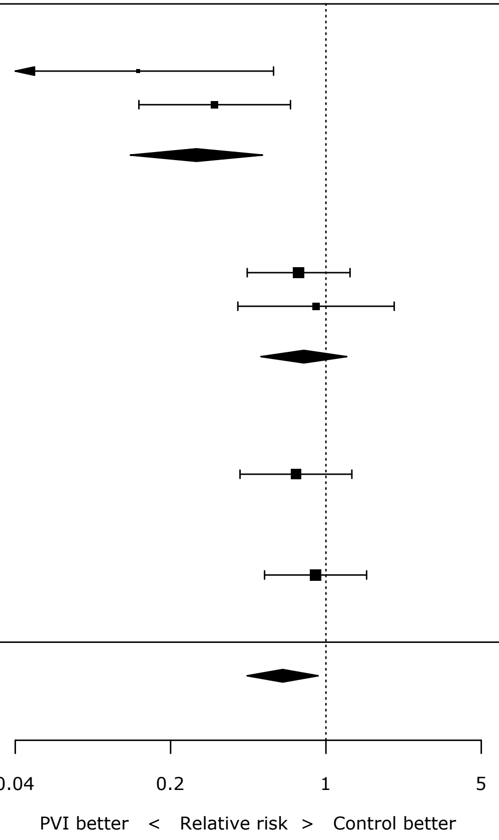

Figure 12

Effect of PVI vs. Non-PVI ablation on AF recurrence at 6 months including the retracted trial OASIS, excluding Di Biase et al

Study and Year	Active Therapy	Active Events	Active N	Control Therapy	Control Events	Control N	Relative risk [95% CI]
CFAE studies							
Chen, 2011	PVI	17	60	CFAE	25	58	0.66 [0.40, 1.08]
STAR AF, 2010	PVI+CFAE	6	34	CFAE	19	34	0.32 [0.14, 0.69]
Random effects model for CFAE studies ($Q = 2.38$, $df = 1$, p for heterogeneity = 0.12; $I^2 = 58.1\%$)							0.49 [0.24, 0.99]
GP studies							
Katritsis, 2013	PVI+GP	18	81	GP	23	78	0.75 [0.44, 1.28]
Mamchur, 2014	PVI	9	41	GP	9	37	0.90 [0.40, 2.03]
Random effects model for GP studies ($Q = 0.13$, $df = 1$, p for heterogeneity = 0.72; $I^2 = 0.0\%$)							0.80 [0.51, 1.24]
FIRM studies							
OASIS, 2016	PVI+FIRM	13	34	FIRM	12	23	0.73 [0.41, 1.31]
HFSA studies							
RADAR-AF, 2014	PVI	18	58	HFSA	19	55	0.90 [0.53, 1.52]
Random effects model for all studies ($Q = 5.34$, $df = 5$, p for heterogeneity = 0.38; $I^2 = 0.0\%$)							0.71 [0.55, 0.90]
p for interaction between study group and effect size = 0.506							p for overall effect = 0.005

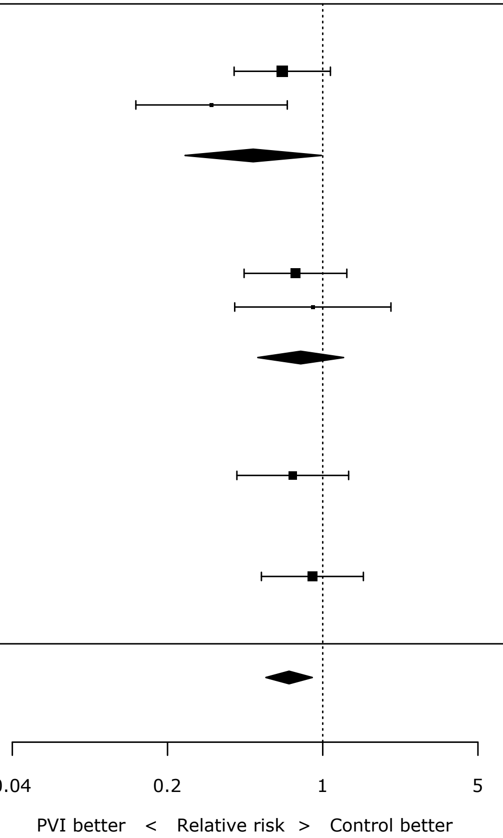

Figure 13

Effect of PVI vs. Non-PVI ablation on AF recurrence at 6 months including the retracted trial OASIS, excluding Katritsis et al

	Active			Control			
**Study and Year**	**Therapy**	**Events**	**N**	**Therapy**	**Events**	**N**	**Relative risk [95% CI]**
CFAE studies							
Chen, 2011	PVI	17	60	CFAE	25	58	0.66 [0.40, 1.08]
Di Biase, 2009	PVI+CFAE	2	34	CFAE	14	34	0.14 [0.04, 0.58]
STAR AF, 2010	PVI+CFAE	6	34	CFAE	19	34	0.32 [0.14, 0.69]
Random effects model for CFAE studies (Q = 5.48, df = 2, p for heterogeneity = 0.06; I^2 = 64.0%)							0.37 [0.17, 0.83]
GP studies							
Mamchur, 2014	PVI	9	41	GP	9	37	0.90 [0.40, 2.03]
FIRM studies							
OASIS, 2016	PVI+FIRM	13	34	FIRM	12	23	0.73 [0.41, 1.31]
HFSA studies							
RADAR-AF, 2014	PVI	18	58	HFSA	19	55	0.90 [0.53, 1.52]
Random effects model for all studies (Q = 9.96, df = 5, p for heterogeneity = 0.08; I^2 = 38.3%)							0.62 [0.44, 0.89]
p for interaction between study group and effect size = 0.523							p for overall effect = 0.009

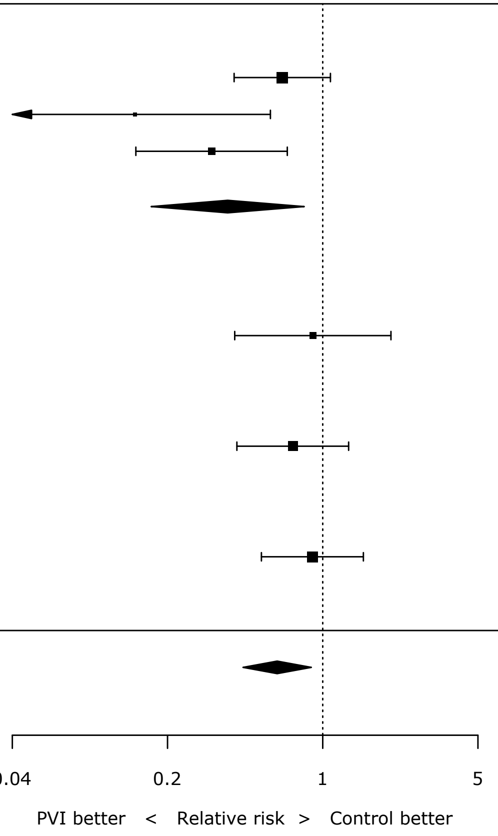

Figure 14

Effect of PVI vs. Non-PVI ablation on AF recurrence at 6 months including the retracted trial OASIS, excluding Mamchur et al

Study and Year	Active Therapy	Active Events	Active N	Control Therapy	Control Events	Control N	Relative risk [95% CI]
CFAE studies							
Chen, 2011	PVI	17	60	CFAE	25	58	0.66 [0.40, 1.08]
Di Biase, 2009	PVI+CFAE	2	34	CFAE	14	34	0.14 [0.04, 0.58]
STAR AF, 2010	PVI+CFAE	6	34	CFAE	19	34	0.32 [0.14, 0.69]
Random effects model for CFAE studies (Q = 5.48, df = 2, p for heterogeneity = 0.06; I^2 = 64.0%)							0.37 [0.17, 0.83]
GP studies							
Katritsis, 2013	PVI+GP	18	81	GP	23	78	0.75 [0.44, 1.28]
FIRM studies							
OASIS, 2016	PVI+FIRM	13	34	FIRM	12	23	0.73 [0.41, 1.31]
HFSA studies							
RADAR-AF, 2014	PVI	18	58	HFSA	19	55	0.90 [0.53, 1.52]
Random effects model for all studies (Q = 9.62, df = 5, p for heterogeneity = 0.09; I^2 = 21.4%)							0.64 [0.48, 0.85]
p for interaction between study group and effect size = 0.570							p for overall effect = 0.002

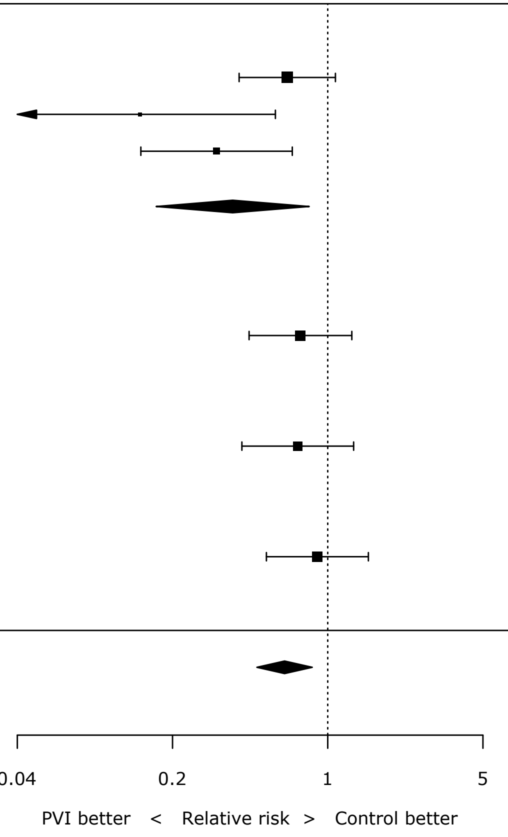

Figure 15

Effect of PVI vs. Non-PVI ablation on AF recurrence at 6 months including the retracted trial OASIS, excluding RADAR-AF

Study and Year	Active Therapy	Active Events	Active N	Control Therapy	Control Events	Control N	Relative risk [95% CI]
CFAE studies							
Chen, 2011	PVI	17	60	CFAE	25	58	0.66 [0.40, 1.08]
Di Biase, 2009	PVI+CFAE	2	34	CFAE	14	34	0.14 [0.04, 0.58]
STAR AF, 2010	PVI+CFAE	6	34	CFAE	19	34	0.32 [0.14, 0.69]
Random effects model for CFAE studies (Q = 5.48, df = 2, p for heterogeneity = 0.06; I^2 = 64.0%)							0.37 [0.17, 0.83]
GP studies							
Katritsis, 2013	PVI+GP	18	81	GP	23	78	0.75 [0.44, 1.28]
Mamchur, 2014	PVI	9	41	GP	9	37	0.90 [0.40, 2.03]
Random effects model for GP studies (Q = 0.13, df = 1, p for heterogeneity = 0.72; I^2 = 0.0%)							0.80 [0.51, 1.24]
FIRM studies							
OASIS, 2016	PVI+FIRM	13	34	FIRM	12	23	0.73 [0.41, 1.31]
Random effects model for all studies (Q = 8.75, df = 5, p for heterogeneity = 0.12; I^2 = 0.0%)							0.63 [0.48, 0.82]
p for interaction between study group and effect size = 0.284							p for overall effect = 0.001

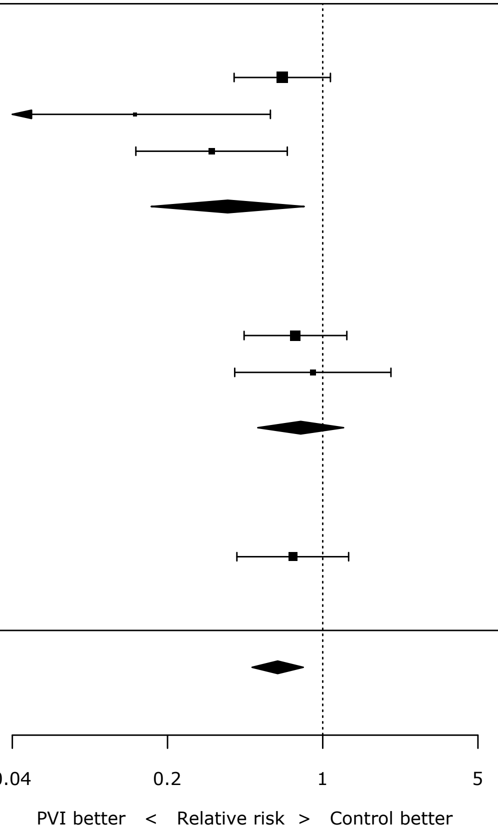

Figure 16

Effect of PVI vs. Non-PVI ablation on AF recurrence at 6 months including the retracted trial OASIS, excluding STAR AF

Study and Year	Active Therapy	Active Events	Active N	Control Therapy	Control Events	Control N	Relative risk [95% CI]
CFAE studies							
Chen, 2011	PVI	17	60	CFAE	25	58	0.66 [0.40, 1.08]
Di Biase, 2009	PVI+CFAE	2	34	CFAE	14	34	0.14 [0.04, 0.58]
Random effects model for CFAE studies (Q = 4.03, df = 1, p for heterogeneity = 0.04; I^2 = 75.2%)							0.35 [0.08, 1.54]
GP studies							
Katritsis, 2013	PVI+GP	18	81	GP	23	78	0.75 [0.44, 1.28]
Mamchur, 2014	PVI	9	41	GP	9	37	0.90 [0.40, 2.03]
Random effects model for GP studies (Q = 0.13, df = 1, p for heterogeneity = 0.72; I^2 = 0.0%)							0.80 [0.51, 1.24]
FIRM studies							
OASIS, 2016	PVI+FIRM	13	34	FIRM	12	23	0.73 [0.41, 1.31]
HFSA studies							
RADAR-AF, 2014	PVI	18	58	HFSA	19	55	0.90 [0.53, 1.52]
Random effects model for all studies (Q = 6.23, df = 5, p for heterogeneity = 0.28; I^2 = 0.0%)							0.73 [0.57, 0.93]
p for interaction between study group and effect size = 0.666							p for overall effect = 0.012

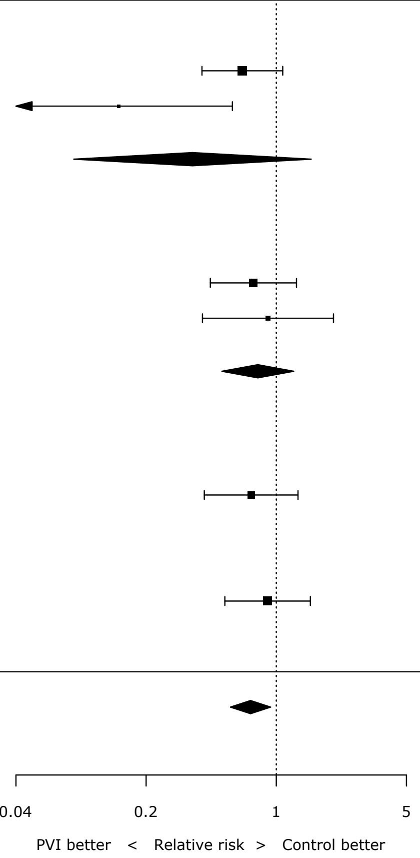

Supplement: Online Data [file mmc1.pdf]
